# Perspectives of Patients and Providers in Using Shared Decision Making in Psychiatry

**DOI:** 10.1007/s10597-021-00856-z

**Published:** 2021-06-27

**Authors:** Natalie J. Maples, Dawn I. Velligan, Eric C. Jones, Erin M. Espinosa, Robert O. Morgan, Melissa A. Valerio-Shewmaker

**Affiliations:** 1 Department of Psychiatry and Behavioral Sciences, UT Health San Antonio, San Antonio, TX USA; 2 Department of Epidemiology, Human Genetics and Environmental Sciences, UT School of Public Health, El Paso, TX USA; 3 National Council on Crime and Delinquency, Austin, TX USA; 4 Department of Management, Policy, and Community Health, UT School of Public Health, Houston, TX USA; 5 Department of Health Promotion and Behavioral Science, UT School of Public Health, San Antonio, TX USA; 6 Department of Psychiatry and Behavioral Sciences, MS7797, University of Texas Health Science Center, 7703 Floyd Curl Drive, San Antonio, TX 78229-3900 USA

**Keywords:** Severe mental illness, Serious mental illness, Shared decision making, Transitional care clinic, Mental health

## Abstract

There is increased interest over the last decade in the use of Shared Decision Making with individuals with serious mental illness to improve engagement in treatment and clinical outcomes. We conducted semi-structured qualitative interviews with 15 individuals with serious mental illness treated in an outpatient transitional care clinic serving people immediately after discharge from a psychiatric hospitalization. Parallel interviews were conducted with a variety of clinical providers (*n* = 9). Using latent thematic analysis, six themes were identified including: (1) Differences in the Use of SDM, (2) Consideration of Past Experiences, (3) Decisional Power Preferences, (4) Use of SDM in Psychiatry Versus Other Areas of Medicine, (5) Dignity and Disengagement, and (6) External Forces Impacting SDM. Implications for clinical practice and research using a shared decision-making approach within this treatment setting are further discussed.

## Introduction

Engagement in mental health services following psychiatric hospitalization is of critical importance for improving the lives of individuals with severe mental illness and reducing long term negative outcomes, such as hospitalization, homelessness and suicide (Velligan et al., 2009; Pasic et al., 2005). People receiving medical care who feel they are playing an active role in their treatment typically have better engagement and outcomes than people who experience themselves as passive recipients of care (Alegria et al., 2014; Stewart, 2001). Although various intervention approaches have been used to increase engagement in outpatient treatment in serious mental illness (Roberts & Bailey, 2011), most methods do not emphasize the importance of the individuals participating in their own treatment decisions. Shared Decision Making (SDM) is an exception. SDM is a structured approach to communication in medicine that frames the interaction as a negotiation between mutual experts (patient and provider) and stresses the balanced use of empirical information and patient preferences and values in decision making (Charles et al., 1997). SDM is compatible with evidence-based medicine in its emphasis on the use of empirical information to make treatment decisions (Montori & Guyatt, 2001), and with the mental health recovery movement in its emphasis on patient-centered care (Drake et al., 2010). Furthermore, the process of recovery from mental health conditions depends on people in treatment taking personal accountability for medical decisions, including those about medications and types of therapies.

SDM is well operationalized, with specific components and clinician competencies detailed in the literature (Campbell et al., 2007; Elwyn, 2005). Several of the fundamental SDM principles aim to help the individual in treatment become more aware a medical decision is needed, offer more than one option from which to choose, communicate the pros and cons of the different options and support the person to make informed choices (Sepucha et al., 2010). A shared decision-making method recognizes the expertise gleaned from both providers and individuals in treatment in making a joint decision. Additionally, research trials demonstrate interventions involving SDM are feasible and well tolerated by individuals experiencing serious mental illness (Deegan & Drake, 2006; Hamann et al., 2011). There is growing evidence in the literature that individuals with severe mental health conditions have positive attitudes towards SDM, a desire to be involved in decision making, and are able to participate in decision making (Hamann et al., 2006, 2007). Research additionally demonstrates that the use of SDM can reduce decisional conflict and improve self-esteem, problem-solving ability, and quality of life in people with schizophrenia (An et al., 2015; Coffey et al., 2019).

### Barriers to SDM Implementation and Dissemination with Serious Mental Illness

Despite the apparent promise of SDM, there is a low level of adoption and implementation of this practice within the delivery of mental health care, relative to other areas of medicine (Pollard et al., 2015; De Las Cuevas et al., 2013; Drake et al., 2010; Hamann et al., 2006). Whether in physical medicine or mental health, efforts to increase patient involvement in decisions about their care face barriers such as overworked physicians, insufficient provider training, deficient medical information systems and physician time constraints (Legare et al., 2008; Towle, 2006). Specific to psychiatry, further complexity is introduced with the potential lack of applicability of SDM due to individual characteristics of people in treatment and inappropriate clinical situations (Solbjor et al., 2013). From the standpoint of efficiency, when SDM is used in prescriber–patient interactions, visits are perceived by prescribers to take more time than does a standard psychiatry appointment (Burton et al., 2010) and doctors cite this as one factor that limits their use of SDM (Legare et al., 2008).

Despite a desire to know more about their diagnosis, treatment options and side effects (Drake et al., 2010; Hamann et al., 2007), individuals receiving psychiatric care report that prescribers often do not provide sufficient information or explain it in an understandable manner (Garfield, 2004; Lorem et al., 2014). Attitudes about the use of SDM have been found to differ by profession, with physicians being more likely than occupational therapists and pharmacists to communicate about the pros and cons of medical issues (Chong et al., 2013). At the patient level, difficulties in using SDM may be due in part to illness-related information processing challenges of the individual in treatment (Mahone et al., 2011; Hamann et al., 2011; Chong et al., 2013; McCabe et al., 2013), suggesting the need for the use of decision support aids and other scaffolding techniques to aid individual preferences in decisions about care (Deegan & Drake, 2006) that may be different from those used for people without a mental health diagnosis. The use of such aids may become an additional barrier due to the further resources required to implement, including time, training in their use, and supplies.

### Facilitators of SDM Implementation and Dissemination in Serious Mental Illness

Evidence supports the use of SDM for individuals with serious mental illness (Hamann et al., 2011; Drake et al., 2010; Deegan & Drake, 2006). Research on SDM in psychiatry has received support through the President’s New Freedom Commission on Mental Health and other important government policy reports supporting the notion that transformation of the mental health service delivery system to promote recovery hinges on treatments that give individuals real and meaningful choices about treatment options (Hogan, 2003; Institute of Medicine, 2001). SDM may be of particular benefit in serious mental illness because treatment plans may not be followed due to factors including dissatisfaction with side effects from antipsychotic and mood stabilizing medications, poor insight into illness and functional and motivational challenges.

For individuals who decline to take medications, SDM may constitute a reasonable approach to remaining engaged with care providers allowing them to take advantage of treatment options such as case management or psychotherapy, while continually re-evaluating the potential role of medication. For individuals in treatment with poor insight or functional challenges, SDM represents a non-threatening approach to clarifying motivations and options for improving quality of life. Despite evidence of cognitive challenges and problems with insight, there is evidence that adults with serious mental illness frequently make competent and prudent decisions (MacDonald-Wilson et al., 2017). For example, in studies of decisional capacity, individuals with schizophrenia (Carpenter et al., 2000) and severe depression (Lapid et al., 2003) performed as well as their non-ill counterparts on measures of ability to provide informed consent following an educational intervention (Carpenter et al., 2000).

Additionally, the Institute of Medicine (IOM) rebutted the belief that this group of individuals should be assumed to have impairment in decisional capacity when stating, “many people with mental illness, indeed, many with severe mental illnesses, are not incompetent on most measures of competency” (IOM, 2006, p. 112). Despite frequent occurrences of behavioral nonparticipation, people with serious mental health conditions express a strong desire to be informed about their illness and treatment options, and to be active participants in their treatment decisions (Velligan et al., 2017; Adams et al., 2007; Arora and McHorney, 2000; De Las Cuevas et al., 2013). Similarly, mental health providers report positive attitudes toward SDM including being more open to discussion with individuals in treatment and increased offering information and choice (Hamann et al., 2007; Seale et al., 2006).

Despite promising data on the use of SDM, the uptake of this practice is poor within services provided to individuals living with serious mental illness (Pollard et al., 2015; De Las Cuevas et al., 2013; Drake et al., 2010; Hamann et al., 2006). The aim of this study was to identify the factors influencing the use of a shared decision-making model in a transitional care clinic providing treatment to people with serious mental illness after the occurrence of a psychiatric crisis or hospitalization.

## Methods

### Design

Given that SDM is a model based on transaction and personal interaction mediated by various kinds of aids or tools (Curtis et al., 2010), the design involved semi-structured interviews to produce data that could be explored inductively regarding the kinds of aids, tools and barriers to effective shared decision making. We conducted one-on-one explorative interviews with individuals in treatment and a variety of clinical providers. Fifteen individuals in treatment and nine providers participated in one-on-one interviews describing their experiences with medical decision making at the Transitional Care Clinic and in comparison to previous treatment settings. Topics addressed during interviews with people in treatment included experiences with prescriber interactions and impact of the SDM process on medication visit outcomes and recovery. Providers discussed experiences using SDM as a practice in general and, specifically about feasibility and utility for individuals seeking care at the Transitional Care Clinic. The interviewer followed a semi-structured interview guide starting with broader questions and ending with more focused discussions on specific topics.

### Setting and Sample

The study site was a Transitional Care Clinic funded by area hospitals, charitable organizations and an 1115 Medicaid Waiver. The clinic provides a wide range of services including medication management, evidence-based psychotherapy and case management for individuals living with severe mental illness recently discharged or deferred from a psychiatric hospital or crisis setting. Severe mental illness is defined as a DSM-5 primary diagnosis of schizophrenia, schizoaffective disorder, bipolar disorder (with and without psychotic features), depression (with and without psychotic features), unspecified psychosis and unspecified mood disorder. As part of an engagement-focused research study conducted from 2014 and 2016 at the Transitional Care Clinic (Patient-Centered Outcomes Research Institute [PCORI]; Contract number: IH-1304-6506), all clinic providers were trained in SDM and continue to be trained in this modality. Provider participants were recruited via email from the first author requesting participation in the study. The nine participating providers included three psychiatrists, one nurse practitioner, one nurse, two psychologists (also served as clinic administrators) and two therapists. Patient participants were recruited during regular medication clinic visits on two separate days in September 2019. Individuals presenting at the clinic were asked by clinic staff about their interest in participating and all had capacity to provide consent. Interviews for patient and provider participants were conducted by the first author and lasted approximately 20–40 min. All interviews were conducted at the clinic and patient participants were paid $30 for their time. The individuals engaged in care interviewed for this study were receiving services from at least one, sometimes more than one, of the interviewed providers. Not all providers from the clinic were interviewed as part of this study.

### Data Collection

Demographics were obtained from both sets of participants including age, gender and ethnicity. The semi-structured interview was developed by the authors (NM and DV) in collaboration with experts in the Department of Psychiatry and Behavioral Sciences, and the stakeholder advisory board of the Transitional Care Clinic. Topics addressed included the patients’ experiences and satisfaction with prescriber interaction, impact of the SDM process on medication visit outcomes and comparisons to previous healthcare experiences. The provider interview guide consisted of questions about experiences using SDM as a practice within the Transitional Care Clinic, including feasibility, and utility. The interview guide began with broad items to allow the participant to begin thinking about their experiences with SDM, what this method entails, their role in the decision-making process, and the information needed to make this approach successful. The interview then moved on to questions regarding how SDM affects participation in sessions and the impact on outcomes and recovery. All questions were open-ended. An excerpt of questions from the patient interview guide can be found in Table 1.

**Table 1:** Excerpt of questions from patient version interview guide

First discussion section—general opening questions
How do you understand shared decision making?
In general, what do you think of the shared decision-making approach?
Are there certain decisions that you want a doctor to make? (Some examples)
Second discussion section—tools or aids utilized
Does your doctor ever use tools during your visits to help you understand what is presented? For example, handouts, videos, or accessing something online during the visit?
Third discussion section—barriers and facilitators
What kind of problems or difficulties do you have in participating in shared decision making?
What was most helpful about shared decision making?
How did you feel about the length of the sessions?
Fourth discussion section—explore for possible changes experienced
In what way did notice differences in how your sessions went compared with other medical office visits?
How do you think SDM impacted how seriously your thoughts and concerns were taken by the provider?
In what way did you feel more or less confident about the choices you made?
Did you feel more like an equal partner in your decisions? (Explain)
First discussion section—outcomes
In what way did SDM help or hurt your recovery?
In what way did SDM help or hurt your chances of continuing mental health treatment?

The provider interview questions were parallel to those used in the patient version, rephrasing for the appropriate interview population. For patient participants who displayed distractibility or difficulty understanding, questions were reworded or repeated. After conducting the fifteen patient and nine provider interviews, it was apparent that similar themes were discussed, and no new themes were emerging. Therefore, saturation was deemed to be reached and no further interviews were conducted. The research study was approved by both the University of Texas Health Science Center at Houston Committee for the Protection of Human Subjects and the University of Texas Health Science Center at San Antonio Institutional Review Board.

### Data Analysis

Interviews were audio recorded and transcribed by research staff and were coded by two authors (NM and DV). Based on grounded theory (Heath & Cowley, 2004)—being cognizant of prior literature on SDM to support the critical examination of the empirical data—we employed latent thematic analysis to analyze this exploratory data (Braun & Clarke, 2006). NVivo 12 software was used to organize the data (NVivo 12, QSR International Pty Ltd, 2018). Each participating group (patient and provider) was analyzed independently and then the group data were combined. Using inductive category development, the codes from all transcripts were thematically clustered to serve as the basis for higher level categories, of which there were 12. Higher order categories included: Individual and Provider Expertise, Choices and Empowerment, Level of Responsibility, Balancing Power, Paternalism History, Dangers of Not Using SDM, Knowledge of Self, Use of Time, Decision Aids, Changes or Trends Over Time, Trust and Honesty, and Differences in Mental Health Treatment. Inductive categorization then allowed for coding the data without trying to fit it into a pre-existing coding frame, or the researcher’s analytic preconceptions (Braun & Clarke, 2006). This flexible method is used to identify patterns of meaning within data among participants and is not allied to any particular framework.

We looked for themes especially relevant to differences in experiences of, and preferences for, SDM (e.g., desired balance of power); satisfaction with provider visits, including length of visits; how previous experiences affect current desire for and use of SDM; and consequences of not using an SDM approach. All codes were then grouped into these categories by the two researchers. Following thematic latent analysis (Braun & Clarke, 2006), the 12 semantic categories were further analyzed at the latent level to identify higher order themes, of which there were six. Any areas of disagreement were resolved by discussion between the researchers until consensus was reached.

## Results

### Sample Characteristics

Of the patient participants, ten were female, four male and one nonbinary. Eight were white Hispanic, six were white non-Hispanic and one was African American. By virtue of attendance at the Transitional Care Clinic, all patient participants met the criteria for severe mental illness as defined previously in Setting and Sample. Participant characteristics can be seen in Table 2. Analysis of patient and provider interviews resulted in six major themes: (1) Differences in the Use of SDM, (2) Consideration of Past Experiences, (3) Decisional Power Preferences, (4) Use of SDM in Psychiatry Versus Other Areas of Medicine, (5) Dignity and Disengagement, and (6) External Forces Impacting SDM.

**Table 2:** Participant characteristics

	Patients (*n* = 15)	Providers (*n* = 9)
Age	*M* = 41.2 (SD = 11.2)	*M* = 48.9 (SD = 9.1)
Gender	4 m, 10 f, 1 nb	4 m, 5 f
Ethnicity	White Hispanic (8)	White Hispanic (4)
	White non-Hispanic (6)	Non-Hispanic white (5)
	African American (1)	African American (0)

### Six Themes

A number of important themes emerged from the research. Exemplary quotes from each theme can be viewed in Table 3.

**Table 3:** Exemplary quotes from six themes

Differences in the use of SDM
“Yeah, we’ll talk about it, and then he’ll tell me about other medications, and then we pretty much kind of make the decision together.” patient
“Well, I was doing good and everything, but all of a sudden, I started getting itches, and I just started feeling weird and stuff, so I was like, ‘Okay, it’s not for me. I don’t think this one’s working, doc.’ And he’s like, ‘Okay. We’ll try something else.’” patient
“I’ve been tempted. Real tempted [to stop all medication]. But the doctor here also says ‘It’s your choice.’ And that’s a real shared decision in saying he doesn’t think it’s a great idea. He really don’t…But, he still let’s me make the final decision.” patient
“I’ve had a lot of really good luck with collaborative decision making in this office.” patient
“But he’s just very determined. If he decides something that’s what it is. He has a lot more power. There’s no partnership.” patient
“The goal is for them [patients] to be equal partners without any power differential or hierarchy.” provider
“By discussing the available and data driven treatments with the advantages and disadvantages for each treatment, it allows the client to be informed of their options for treatment and allows them the opportunity to give input on how they want to be treated?” provider
Consideration of past experiences
“I was detained for, I felt for being honest… I told her exactly how I felt…and I ended up being detained and hospitalized. And I’m like, Well, the hell with this. I’m not gonna talk to these people ever again.” patient
“It’s terrible. They don’t even care. They just give you these prescriptions, thank you, bye-bye.” patient
“I’ve been here a while and it’s not fun, changing from one doctor and starting over. And I don’t want to do that, but I do want my doctor to understand me. I want to because the feeling of a panic attack is horrible. He [the doctor] doesn’t listen.” patient
“It didn’t start off well. I was very angry. I was angry with the cops, I was angry with everybody and angry when I came in here. I came into the clinic very angry and did not want to be here. It was a forced situation. patient
“A lot of them [patients] said we didn’t know this was something that we should do because for so long we were told that we were unable to make decisions.” provider
“Hearing the patient’s point of view really opened my eyes to how they were disempowered and how all the control was essentially put on someone else in their past.” provider
“I’ve seen patients surprised and really engaged in thinking about stuff that they weren’t expecting to be thinking about.” provider
“A lot of them shied away from *having input* and a lot of them said that’s not my place. I should never tell a doctor what to do.” provider
“Learning SDM practices takes time and practice for those who were trained to be more paternalistic.” provider
Decisional power preferences
“I think it should be a mix of both…Because you know what’s best for yourself, and then, well, the doc knows what’s best for you medically through her license. So I think it should be both. It’s a good thing. There’s a balance there.” patient
“No—I don’t think so. I guess because of what did happen to me and that caused me to be here…I think it should still be both because if it was more one-sided—I don’t know how that would work because that’s giving more power to the other person over your life. I think it should still be discussed by both parties equally.” patient
“The doctor should have slightly more because he’s the book smart. He’s done all the education to be the doctor.” patient
“…because I say of course he knows better than me. I’m gonna say this and that. Who am I? I’m just there, so I come here for the help, you know. And that’s it.” patient
“We should have < 50%, unless the patient is exhibiting problems with decisional capacity at that time.” provider
“It may be something like the patient has 65%, doctor has maybe 25%, and then other has 10%, which could include people like family members.” provider
Use of SDM in psychiatry versus other areas of medicine
“It [SDM approach] helps a lot because I never talked about my past traumas before.” patient
“It helps build a trust and respect. I’m not like a paycheck or salary. I respect them [providers] more because they show respect to me.” patient
“It [SDM] offered me an opportunity to assess where I was headed and choose if I wanted to return to the path that I’d already set out for myself, or veer off on another course.” patient
“It [SDM] makes patients want to participate in the process and feel engaged in the process. So I think it probably increased appointment adherence and prescribed treatments, which results in improved outcomes.” provider
“The prescriber has the opportunity to build trust with the patient through the SDM process. When the patient is informed about their treatment options, they are more inclined to engage in treatment.” provider
Dignity and disengagement
“…with regards to medication, there was one time when I introduced the idea of going off medication and he [prescriber] was extremely opposed to the idea of me going off medication…I began experimenting with going off the medication on my own rather than doing so in an observed environment. So I stopped wanting to come in for mental health treatment, which turned into its own snowball of bad feelings. And really hurt. Then when things became a big problem for me, I wasn’t in an environment where I could be assisted, and so by the time that I returned to that environment, it was an emergency situation.” patient
“The doctors that I had seen, she pretty much just made the decision for me, whenever I was saying I wasn’t feeling good, or whatever, trying to decide what medication to take. She pretty much just made the decision. I don’t really like that clinic and I didn’t go back.” patient
“You need to take me into consideration because I know my body.” patient
“Sometimes I wish that, whatever doctor that I’m at, I wish that they could just feel what I’m feeling for just a minute so they could just have a little taste of what I go through.” patient
“I really deeply believe in the importance of patients feeling autonomous, feeling that they are the main person guiding their life.” provider
External forces impacting SDM
“Yes, when symptoms, like impulsivity make me forget the long-term goal.” patient
“…there’s an ability for, kind of, a circumnavigation. Like if I’m starting to veer off course there’s a way of just circumventing that. I probably wouldn’t be going back to school in January if she hadn’t done that [made the decision].” patient
“Not in my experience. There is never a time when the doctor should have more say than the patient.” patient
“If somebody is talking about self-harm…in those moments the doctor needs to step in, in a much more directive way.” provider
“For example, I’ll have patients come in and say, ‘I want to do this.’ And I’ll say, Well, I don’t completely y agree with it, although there is no absolute contraindication. These are the risks associated with it. It’s important to me that you accept the risks.” Provider
“When there’s an absolute contraindication, then I say, ‘There’s an absolute contraindication to this and I won’t prescribe it.’” provider

#### Differences in the Use of SDM

A primary reason in attending appointments at the Transitional Care Clinic is to receive medication for mental health conditions, although many other treatments may be provided in addition to medication management. Specific to discussions of medication, the majority of comments from both patient (13/15) and provider (9/9) participants support the active provision of options including, research data, weighing pros and cons, and collaborative communication. Discussions around medication occur in most medication follow-up visits, even if no changes are needed or made. One patient participant recalled wanting an increase in medication, but that request was not fulfilled. However, a joint discussion still transpired. Prescribers typically offered options each time a medication or therapy was not satisfactory to the individual in treatment, whether due to side effects, potency, or other reasons, and patients were pleased with this communication method. Providers and those engaging in care concur that the final decision on taking medication and deciding which medication to take is the responsibility of the person in treatment, although the clinical provider is heavily relied upon for their expert knowledge. The respect given to provider knowledge was apparent from the vast majority of patient participants (12/15). Three patients described the experience of not feeling like a partner in decision making at the Transitional Care Clinic, recounting a lack of perceived power and an absence of a give-and-take conversation around treatment. All interviewed providers endorsed seeking to use a SDM approach, although none endorsed explaining the actual concept or methodology of SDM with patients. Decision aids (media or methods that inform patients about treatment options) are not regularly used by any interviewed providers to assist individuals in treatment in making decisions, and only three patient participants endorsed a positive attitude about their potential use in mental healthcare.

#### Consideration of Past Experiences

All patient participants discussed experiencing any number of severe symptoms, such as psychotic episodes, debilitating depression, attempts at self-harm, and brief or long-term hospitalizations. Many (11/15) described previous negative experiences during psychiatric hospitalizations where they perceived having no input on anything, for example, daily schedule, medications taken and discharge date. These experiences created a perception of having no power in these situations. Similarly, several providers (4/9) discussed creating a disempowered group of patients because of these types of negative inpatient experiences where decisions were made by providers without patient input. This has the potential to teach dependence on providers and less confidence in one’s ability to make autonomous decisions. Providers further explained these negative, and often traumatic, experiences with no opportunity for input or perceived control shaped how people in treatment believe they may or may not participate in their healthcare management. They also refer to health disparities often present in the population of people living with severe mental illness, including economic disadvantage, lack of access to healthcare and lower levels of education, as being interrelated to patients’ perceptions and understanding of mental healthcare. Providers acknowledged patients may not “know how to ask to be an equal partner” in their mental health treatment because they have essentially been trained not to do so. Comparably, most providers (8/9) also discussed differences in their own education and background influencing their beliefs about a SDM approach to care. Specifically, there is a difference of opinion amongst providers as to the extent to which SDM is taught to mental health professionals during their formal education. Additionally, the collaboration is more required for some job roles and providers acknowledged the use or uptake of a SDM approach may be generational, in that more newly trained providers may have received more education on this communication method.

#### Decisional Power Preferences

Both groups of participants agreed the person in treatment and provider should share decision-making responsibility and many patients (7/15) cited “50/50” as the appropriate balance of decision-making accountability. Most providers conveyed the person in treatment should have more than 50% of the decisional authority (i.e. 60/40, 75/25), although two providers stated the situations and decisions varied too much to approximate a percentage. Two providers also introduced the importance of significant others in the patients’ lives, such as family members, who are part of the onus to providing input for health-related decisions. While most patient participants prefer nearer to an equal partnership, a small number (3 of 15) preferred to concede power to the provider. These three were all white Hispanic females.

#### Use of SDM in Psychiatry Versus Other Areas of Medicine

The use of SDM in psychiatry was viewed as more important than its use in non-mental health settings. Many providers (6/9) cited the lack of a specific test or scan to diagnose and treat ailments. “We don’t have an x-ray machine.” In these situations, patients participating as their own expert is of increased value. Individuals in treatment described the importance of sharing in decisions as the precursor to a trusting relationship that fosters honesty and recovery. Several patients said they can talk to their current provider about topics they have never discussed previously with other providers—for example, repeated childhood trauma. This aligns with providers’ reports that sharing information is the best way to proceed with appropriate treatment options. The information on how someone is feeling what they have experienced informs the process of how physicians and therapists make treatment decisions. Four providers mentioned pre-established guidelines for medication and therapies based on their knowledge of the case. If individuals in treatment do not provide accurate or honest information, providers cannot put those protocols into action. In physical medicine, there is often a clearly superior treatment option, for instance in cancer treatment, but this is typically not the case in mental health treatment—thus increasing the reliance on patient involvement. Providers and patients agree it may take more time upfront to engage in discussions that are essential to a SDM approach, but all reported this communication practice saves time later. For example, providers mentioned that extra time spent on discussions within the office may help reduce: (1) extra phone calls to clinic staff because details were omitted, (2) symptom recurrence and possible hospitalization, and (3) patient’s experience of unexpected side effects. Likewise, providers (9/9) did not believe using a SDM approach uses more resources over the course of treatment. Several providers mentioned a higher likelihood of individuals showing up to appointments as a potential cost saving aspect of using SDM.

#### Dignity and Disengagement

Almost half of the interviewed patients (7/15) describe times, either in this clinic or in previous experiences, where they did not feel their opinions were taken into consideration and stopped seeking treatment or taking medication altogether. These same individuals described specific times where they requested a change in medication or other treatment and the provider did not consider their request; there was no attempt at discussion. Three of these seven individuals describe stopping all treatment, which led to an increase in symptoms, suicide attempts and/or hospitalization. Patients (7/15) and providers (4/9) who engaged in this discussion with the author agree it is better to taper off of a medication or other treatment while remaining engaged with care providers. Based on the information gleaned in the interviews, possible consequences of not allowing patient input is their disengagement from the mental health system, self-harm, rehospitalization, or other negative outcomes. While decisions are sometimes made for individuals in treatment, many (10/15) described being their own expert and believe their personal knowledge must be considered, even if not pursued. There is a dignity in risk. While patients understand there may be negative consequences to reducing or stopping treatment, they want partnership in attaining their health goals, sometimes in opposition of what the providers believes is best practice.

#### External Forces Impacting SDM

The majority of patients and all provider participants support the notion of SDM and its use with individuals in a psychiatric setting. However, almost all indicated times when a more paternalistic approach is needed. The need for an authoritative style was linked to recurrent or worsening symptoms or fear for the safety of the person receiving treatment, including intent of suicide or homicide. All providers and all but one patient agreed the clinical provider, often the physician, carries the weight of making decisions for the individual in care when their decisional capacity is impaired due to an “acute exacerbation of symptoms.” Likewise, providers exert a responsibility to their own clinical licenses and training that may be in opposition to what patients want. Most providers (7/9) listed following clinic (or systemic) rules as a responsibility (i.e., adhering to a rule restricting the prescribing of benzodiazepines) that may negatively impact the provider-patient relationship because the decision is out of their hands. Providers and patients agreed a discussion can still transpire that can minimize the negative feelings associated with not having a full or real choice.

## Discussion

This is the first evaluation of the perceptions and preferences of individuals living with serious mental illness and their providers regarding SDM in a clinic serving this population immediately following a psychiatric crisis or hospitalization. Results of this qualitative study suggest that patients and providers value SDM, understand its application, and believe it is related to better outcomes. More concordance than disagreement exists between the two groups of participants throughout the six themes. Both sets of participants believe the use of SDM in the field of psychiatry is valuable and necessary, including open and collaborative communication. Providers and people receiving care concur that the final decision on taking medication and deciding which medication to take is the responsibility of the person in treatment, although the clinical provider is heavily relied upon for their expert knowledge. Both sets of participants also acknowledge that previous negative experiences unintentionally disempower individuals receiving mental health care and there is room for improvement in enhancing recovery-oriented care within mental health. There is much variation in the amount of decisional power a person in care may seek, whereas providers demonstrated less variability in their answers about this topic. Specifically, individuals in care reported opinions ranging from almost none to almost all in respect to the amount of decisional power desired in mental health decision-making. This may reflect different cultural backgrounds, age, or any number of previous experiences in healthcare. In addition to these mostly concordant views within the six themes, several important topics are evident and can inform future clinical care and research in this field.

### Implications for Clinical Practice

The study supports the need to ensure that SDM is a routine practice in psychiatric care. Study participants made comments which are supported by research suggesting that a continued paternalistic approach in medical decision-making continues to socialize individuals into the role of patient rather than equal partner (Murgic et al., 2015). People receiving treatment and providers identified potential serious consequences including, disengagement and abruptly terminating treatment, when individuals do not participate in decisions and do not feel heard. Studies of SDM link increased patient involvement to improved treatment adherence and quality of life, while lack of their involvement is related to lower concordance with treatment plans, patient satisfaction, and overall health outcomes (Sepucha & Mulley, 2009). The reports that SDM improved show rates and reduced calls in between appointments is important to those receiving care, providers and the agencies for which they work. Research continually demonstrates no shows increase overall healthcare costs and reduce the gains made from treatment (Kheirkhah et al., 2016; Berg et al., 2013).

Both individuals in treatment and providers in this study assert that trust in the clinical provider and patient honesty is strengthened by the use of SDM. People receiving mental health care are more likely to share personal information needed to inform treatment options. Research supports that a trusting relationship with a clinical provider can strengthen alliance and prevent crises or other serious negative outcomes (Arnow & Steidtmann, 2014; Howgego et al., 2003). To improve partnerships, clinical providers can offer options and have a conversation, even when yielding to the individual’s request is not feasible. As part of this conversation, providers must consider there is a certain dignity in risk-taking, even when not medically supported (i.e. getting off medication). Providers can state the pros and cons of certain decisions, but the final decision is left to the individual in treatment, assuming decisional capacity is intact. This research demonstrates a high level of agreement between providers and patients on times when providers may need to have more power and this has clinical implications for discussions that can occur at the beginning of treatment, or prior to an acute exacerbation of symptoms. There is opportunity to discuss potential situations such as these in the informed consent process for incoming/new patients, thereby increasing open communication at the onset, including gathering information on what the patient might want should decreased capacity occur temporarily.

Although there is less of an emphasis of SDM in mental health care (Pollard et al., 2015; De Las Cuevas et al., 2013; Drake et al., 2010; Hamann et al., 2006), the findings of this study suggest the use of a SDM approach is more important in this filed compared to traditional somatic care. Psychiatric care is dependent on the nature of communication and this is instrumental in whether care is deemed effective. When you do not have a clearly superlative treatment, often the case in psychiatry, the reliance on SDM becomes more important (Morant et al., 2016). As mental health treatment increasingly reaches toward a recovery-oriented system of care, Barry and Edgman-Levitan (2012) argue that SDM is the highest form of patient-centered care.

### Implications for Research

There is a need for more effectiveness studies on the influence of training in SDM practices for both individuals in treatment and providers. The former may benefit from learning about the methodology of SDM to level the playing field. That is, more explicitly explaining the concept, what it means, the benefits and the responsibilities involved. Likewise, in the increasingly diversified field of healthcare, practitioners enter their jobs with differing amounts of education on SDM practices. The effect of attempts to improve providers’ knowledge on this practice and implementation of SDM within the healthcare field remains insufficient (Legare et al., 2018). Implementation science research on the uptake of SDM strategies into organizational culture and climate is needed. These larger scale studies can test and evaluate different mechanisms of incorporating SDM into routine mental health care. The results from such trials can also inform public policy and legislation as the role of SDM is part of the larger health care reform conversation. Additionally, investigation of racial and ethnic differences in decision-making preferences can better inform clinical providers on how to individualize communication on decisional authority. While this study has too small a sample to draw conclusions on demographic information, the tendency for white Hispanic females to cede their autonomy needs further investigation. In somatic medicine, most interventional research on SDM has focused on decision aids to help people in treatment build their preferences or to facilitate any kind of engagement (O’Connor et al., 2011) with far fewer attempts in the use of this strategy in mental health (Drake et al., 2010). The use of decision aids was not endorsed in this study but may be important in empowering individuals engaged in mental health care to make more independent and informed choices.

While many important themes and implications for future work were found from this research, several limitations are present. All interviews were performed in one clinic with individuals who spoke English. Interviewing participants in different areas and those who speak other languages may have extended the variability of responses. Only one of 24 total participants were African American limiting the generalizability of these findings. Additionally, the concept of SDM was new to several of the patient participants and it is possible that our explanations influenced their answers.

In conclusion, shared decision making was viewed positively by all providers and most individuals in treatment, although there is a wide range of opinions in the amount of power either person should have in medical decision making. Some people receiving care are more likely to cede their control to the provider, resulting in the need for providers to adapt their approach to presenting information and eliciting patient engagement. Trust and honest communication is foundational to improved outcomes for individuals living with serious mental health conditions and shared decision making is a tool to foster necessary engagement. Dignity of risk is essential for individuals engaged in mental health care and the danger of not providing this opportunity is disengagement from treatment altogether. SDM is a practice with the potential to advances the goals of mental health transformation identified by state and federal stakeholders as being necessary for improved outcomes for those living with mental health conditions.
